# Dissecting the role of transforming growth factor-β1 in topmouth culter immunobiological activity: a fundamental functional analysis

**DOI:** 10.1038/srep27179

**Published:** 2016-06-02

**Authors:** Pengzhi Qi, Congxin Xie, Baoying Guo, Changwen Wu

**Affiliations:** 1 National Engineering Research Center of Marine Facilities Aquaculture, Marine Science and Technology College, Zhejiang Ocean University, Zhoushan 316004, China; 2 College of Fisheries, Huazhong Agricultural University, Wuhan 430070, China

## Abstract

Transforming growth factor-*β1* (TGF-*β1*) has been proven to function primarily in mammalian immunobiological activity, but information regarding the immune role of TGF-*β1* in teleosts is limited. In the present study, we describe the cDNA cloning and characterization of the TGF-*β1* molecule in the topmouth culter. TGF-*β1* is highly expressed in immune-related tissues of the culter, including the thymus, head kidney, and spleen. The recombinant culter TGF-*β1* (*cTGF*-*β1*) was successfully expressed and purified *in vitro*, and the effects of *cTGF*-*β1* on the mRNA expression of pro-inflammatory cytokines, such as TNF-*α* and IL-*1β*, in the absence or presence of LPS was determined in culter peripheral blood leukocytes. *cTGF*-*β1* was found to have bipolar properties in inflammatory reactions. Additionally, to assess the immune role of teleost TGF-*β1 in vivo*, the expression of TGF-*β1* in the culter thymus and spleen tissues induced by poly I:C were also examined. The expression of TGF-*β1* was obviously up-regulated, as shown in the cell lines. However, the peak time of *cTGF-β1* expression in the cell lines occurred significantly earlier than in the organic tissues under the same inducer, suggesting that the response of the teleost TGF-*β1* molecule to exogenous infection depends on a more complicated signalling pathway *in vivo* than *in vitro*.

Transforming growth factor-*β* (TGF-*β*) has been studied largely over the past few decades and has been demonstrated to be an important multifunctional cytokine involved in the regulation of cell proliferation, differentiation, survival, migration and apoptosis under physiological and pathological conditions^1^. TGF-*β* is initially released from macrophages, fibroblasts and platelets^2^ as a small complex termed ‘latent TGF-*β*’, which consists of mature TGF-*β* associated with the latency-associated protein (LAP)^3^. Before TGF-*β* can perform its function, the active TGF-*β* must be separated from the LAP and the binding protein; subsequently, the cytokine exerts its effects via the receptor-*Smad* signal transduction pathway^4^.

Vast evidence indicates that TGF-*β* plays a critical immunoregulatory role in mammals both in the innate and adaptive immune pathways^5^. In general, TGF-*β* inhibits T cell proliferation by blocking interleukin-2 production and cyclin expression^1^ and exerts multiple stimulatory effects on B cells, natural killer cells and dendritic cells, including the activation of these cells and the regulation of chemotaxis^6^,^7^,^8^. TGF-*β* has also been shown to regulate the active and inactive states of monocytes and macrophages under specific conditions^9^. Finally, TGF-*β* can up-regulate the expression of the fibronectin receptor by monocytes^10^,^11^,^12^, making TGF-*β* a potent chemoattractant^13^,^14^.

Three TGF-*β* isoforms, TGF-*β1*, *β2* and *β3*, have been identified in mammals, among which TGF-*β1* plays a primary role in immunobiological activity. The TGF-*β1* isoform is also the most studied in non-mammals, and has been cloned and characterized in several fish species, including carp^15^, hybrid striped bass^16^, sea bream^17^, grass carp^18^ and goldfish^3^. These studies provided new evidence for use in interpreting the immunoregulatory mechanism of the TGF-*β1* gene in fish species. However, the data pertaining to the functional analysis of this cytokine are still very scarce and fragmentary, and the functional role of TGF-*β1* in fish immunoregulation is still unclear.

*Culter alburnus*, or the topmouth culter (hereafter referred to as ‘culter’), is widely distributed throughout large rivers, reservoirs and lakes in China. Over recent decades, this fish species has become one of the most commercially important freshwater cultured fish species in China. Unfortunately, outbreaks of infectious diseases in freshwater cultured culter species have recently become a major bottleneck in the culter fishery industry. These fish appear to be vulnerable to various pathogens, such as bacteria, viruses and parasites. Thus, finding new clues regarding culter immunomodulation is critical in explaining the susceptibility of this species to pathogens.

Despite the crucial role of TGF-*β* in immunity, the relevant information pertaining to this cytokine in culter remains largely lacking. In the present study, we report for the first time the cloning of TGF-*β1* from culter (*cTGF-β1*), followed by the expression and purification of the TGF-*β1* protein *in vitro*. Furthermore, the effect of *cTGF-β1* on the mRNA expression levels of pro-inflammatory cytokines, including TNF-*α* and IL-*1β*, in the absence or presence of LPS was determined in culter peripheral blood leukocytes (PBLs). The TGF-*β1* molecule showed bipolar properties in the inflammatory reaction. Additionally, the expression levels of the TGF*-β1* molecule were up-regulated in both the culter thymus and spleen tissues after induction with polyinosinic-polycytidylic acid (poly I:C), although TGF*-β1* appeared to be more sensitive to poly I:C induction in the thymus than in the spleen. Taken together, the results presented in this study will help to improve our understanding of the role of TGF-*β1* in teleost immunobiological activity.

## Results

### Molecular cloning and characterization of *cTGF*-*β1*

**Figure 1:**
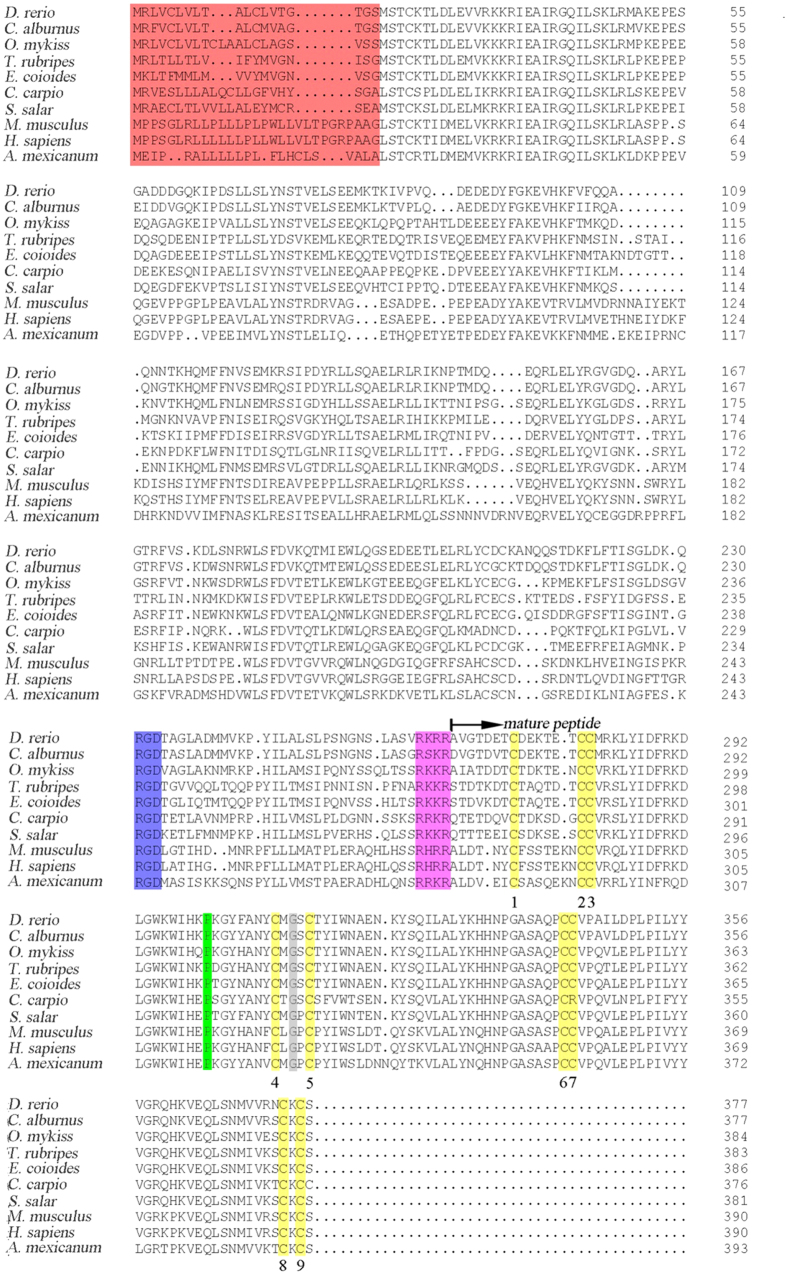
Alignments of the deduced amino acid sequence of *cTGF-β1* with its homologues in other species. Numbers on the right of the sequences indicate the amino acid positions. Gaps are noted with hyphens. The mature peptide portion is noted with the arrow. The signal peptide region, RGD integrin-binding site, RRKR cut site, nine conserved cysteine residues, and conserved proline and glycine at positions 36 and 46 of the mature peptide are marked in red, blue, brown, yellow, green and grey, respectively. The GenBank accession numbers of the detected species are as follows: *D. rerio* (NP_878293.1); *C. alburnus* (AIS23633.1); *O. mykiss* (NP_001268295.1); *T. rubripes* (BAM44873.1); *E. coioides* (ACV96791.1)*; C. carpio* (Q9PTQ2.1); *S. salar* (ACN11294.1); *M. musculus* (NP_035707.1); *H. sapiens* (NP_000651.3); and *A. mexicanum* (ABX24523.1).

The culter TGF-*β1* cDNA (GenBank accession no. KJ725122) was assembled by cDNA cloning based on 3′- and 5′-RACE. The full length *cTGF-β1* cDNA was 2175 bp, including a 1134 bp opening reading frame, a 529 bp 5′ untranslated region and a 512 bp 3′ untranslated region (Fig. S1). The putative *cTGF-β1* sequence, which consisted of 377 amino acids containing the precursor region and mature region, had a molecular weight of approximately 43.21 kDa. An alignment analysis showed that some structural domains that are conserved in mammals and other fish species was also present in the *cTGF-β1* molecule, including a characteristic RGD integrin-binding site and a RRKR cut site in the precursor region, nine cysteine residues allowing for the formation of inter-chain and intra-chain disulfide bonds, the C-terminal cysteine knot in the mature peptide, and the conserved proline and glycine residues in the mature peptide, which are the distinguishing hallmarks of the TGF superfamily (Fig. 1).

**Figure 2:**
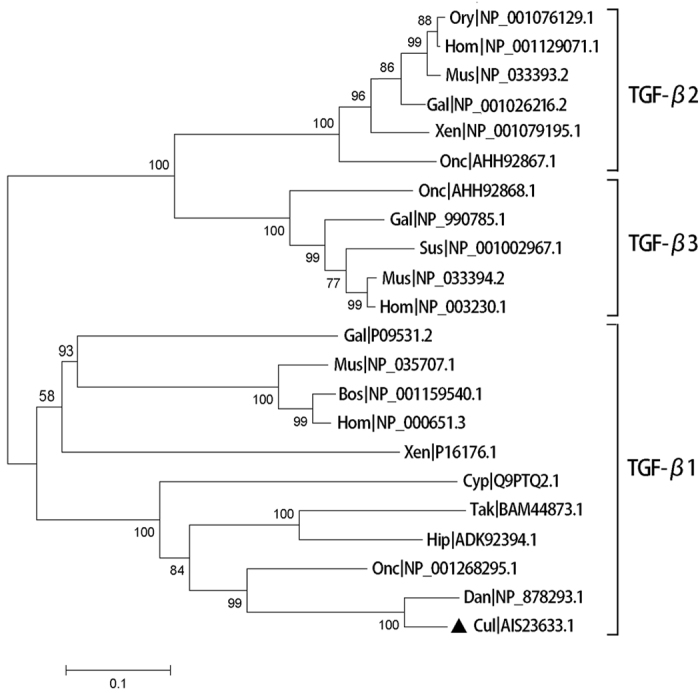
Phylogenetic analysis of *cTGF-β1* with other isoforms based on the neighbour-joining method. *cTGF-β1* is indicated with a black triangle. The numbers near each branch represent the bootstrap values obtained with 10,000 replicates. Abbreviations of the species presented in the figure are as follows: Tak, *Takifugu rubripes*; Onc, *Oncorhynchus mykiss*; Dan, *Danio rerio*; Hip, *Hippocampus kuda*; Cul, *Culter alburnus*; Cyp, *Cyprinus carpio*; Xen, *Xenopus laevis*; Mus, *Mus musculus*; Bos, *Bos taurus*; Gal, *Gallus gallus*; Hom, *Homo sapiens*; Ory, *Oryctolagus cuniculus*; Gal, *Gallus gallus*; and Sus, *Sus scrofa*.

In the phylogenetic tree, *cTGF-β1* first clustered with its zebrafish and rainbow trout homologues (Fig. 2), with which *cTGF-β1* shared the highest sequence identity (91% and 69% amino acid identity, respectively), suggesting that *cTGF-β1* had the closest phylogenetic relationship with zebrafish TGF*-β1* and rainbow trout TGF*-β1*. Various teleost TGF*-β1* molecules clustered into one distinct branch and were subsequently grouped with their congeners from frog, chicken, and mammals to form one large branch, which shared a more distant polygenetic relationship with the TGF-*β2* *s* and TGF-*β3s* of fish, chicken, frogs and mammals (Fig. 2).

### Tissue distribution of cTGF-*β1*

**Figure 3:**
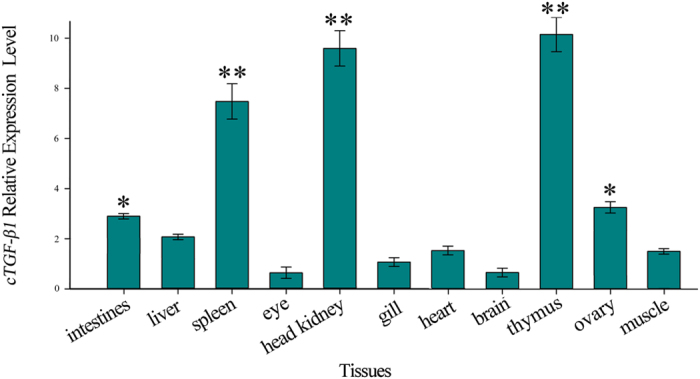
Tissue distribution of TGF*-β1* molecule in culter fish. The results are represented as the mean ± SEM of 8 individual fish. The data were compared relative to *β*-actin (endogenous control) and were normalized to the expression in the gill.

Eleven tissues were analysed using qPCR assays to determine the *cTGF-β1* tissue distribution profile. As shown in Fig. 3, *cTGF-β1* mRNA was constitutively expressed in all detected tissues. However, the expression levels varied significantly among different tissues. The *cTGF-β1* transcript was most highly expressed in the thymus, head kidney, and spleen (surpassing 7-fold relative expression levels compared to the gill), to a lesser extent in the ovary, heart, liver, muscle, intestine and gill (from 1- to 3-fold relative expression levels compared to the gill), and at relatively low levels in the brain and eye (less than 1-fold relative expression levels compared to the gill).

### Construction, expression and detection of the c*TGF-β1* recombinant protein

**Figure 4:**
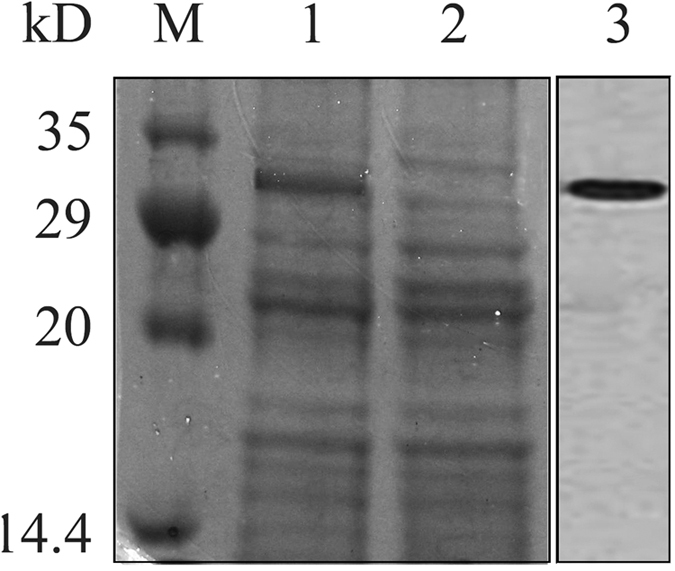
Expression of the mature peptide portion of *cTGF-β1 in vitro*. SDS-PAGE and western blot results using tissue from two testes. Each individual testis are indicated by the black frame. M, protein molecular weight marker; lane 1, lysates of transformed bacteria after induction with IPTG; lane 2, lysates of bacteria transformed with the non-inserted control; lane 3, western blot analysis of recombinant *cTGF-β1* using an anti-6 × His tag mouse antibody.

The mature peptide portion of the deduced *cTGF-β1* amino acids sequence was cloned into the pET32a plasmid with the addition of the BamHI and XhoI cutting sites to construct the expression vector *pET32a-cTGF-β1*. The conditions were optimized, and the fusion protein was efficiently expressed and subsequently purified. An SDS-PAGE analysis showed a distinct protein band of 33.88 kDa, which corresponded to the calculated molecular weight of the recombinant protein after IPTG induction compared to the empty vector control. A western blot analysis indicated that the bacterially expressed protein showed a predominant band with an estimated molecular weight of 33.88 kDa (Fig. 4). After purification of the protein with a commercial kit, no more than 0.1 EU endotoxin residues remained in 1 ml of the purified cTGF-β1 isolate.

### Effects of c*TGF-β1* on the mRNA expression of pro-inflammatory factors in PBLs

**Figure 5:**
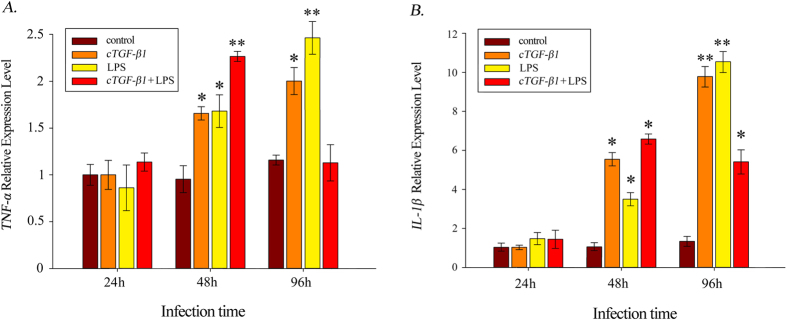
Transcript expression analysis of the pro-inflammatory factors (**A**) TNF-*α* and (**B**) IL-*1β* with the induction of *cTFG-β1*, LPS, or *cTFG-β1* and LPS. The data presented (mean ± SEM, N = 4) are the representative results of three individual experiments. (*) Indicates statistical significance (P < 0.05).

To investigate the role of *cTGF-β1* in the inflammatory reaction, PBLs were isolated and cultured with 100 ng/ml of *cTGF-β1*, 10 μg/ml of LPS, or both of these substances for 24 h, 48 h and 96 h before the RNA was extracted. The variations in the expression levels of TNF-*α* and IL-*1β*, which both attach to pro-inflammatory factors, were chosen for examination. As shown in Fig. 5, these two factors showed expression profiles that were essentially identical after induction. At hour 24, the expression levels of TNF-*α* and IL-*1β* under the three types of induction all showed no significant difference compared to the controls. Subsequently, the expression levels sharply increased, reaching significant levels at hour 48. However, the dual induction with *cTGF-β1* and LPS seemed to promote the expression of TNF-*α* and IL-*1β* much more than either *cTGF-β1* or LPS alone. Notably, the expression of TNF-*α* increased to a highly significant level at hour 48 under the dual induction. The expression levels of TNF-*α* and IL-*1β* continually increased and peaked at hour 96 with induction by either *cTGF-β1* or LPS. By contrast, the expression of these two factors showed noticeable, sharp decreases with the dual induction of *cTGF-β1* and LPS after hour 48; this was especially the case for the expression level of TNF-*α*, which decreased to an undetectable level at hour 96.

### Induction of c*TGF-β1* expression by poly I:C

**Figure 6:**
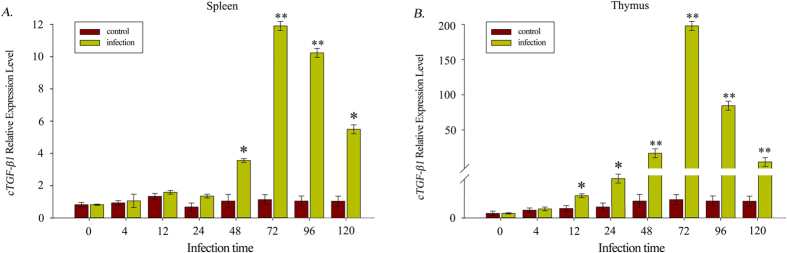
Expression analysis of the cTGF-β1 transcript in the spleen (**A**) and the thymus (**B**) after induction by poly I:C. The values depicted in the graphs are the mean ± SEM of 4 individual fish. (*) Indicates statistical significance (P < 0.05).

To assess the immunological role of TGF*-β1 in vivo*, the expression of this cytokine was examined in two immune-related tissues via induction by poly I:C, which was presented as a viral analogue. As shown in Fig. 6, the induction by poly I:C significantly up-regulated the expression of TGF-*β1* in the thymus and the spleen. The TGF-*β1* expression levels in these two culter tissues both peaked at hour 72 (11.8-fold and 185.6-fold in the spleen and thymus, respectively) and subsequently decreased gradually. However, *cTGF-β1* showed a different sensitivity to poly I:C between the two tissues: in the thymus, *cTGF-β1* appeared to be more sensitive to poly I:C than in the spleen, as the expression of *cTGF-β1* was significantly up-regulated at hour 12 in the thymus compared to hour 48 in the spleen.

## Discussion

In the present study, the full-length cDNA of TGF-*β1* in culter was cloned for the first time, and the amino acid sequence of *cTGF-β1*, which contains 377 residues, was deduced. The alignment analysis showed that all structural domains were strictly conserved in all detected TGF-*β1* counterparts from various species. Phylogenetically, *cTGF-β1* was closely related to its homologue in other vertebrates. All of these results indicated that the teleost TGF-*β1* molecule may be functionally similar to that of higher vertebrates. Indeed, the TGF-*β* system has been shown to be highly conserved; the ligands, receptors and signalling molecules associated with this system can be traced back to arthropods, suggesting that the TGF-*β* system is over 1 billion years old^19^.

The tissue distribution of *cTGF-β1* showed a ubiquitous expression pattern. This result was in accordance with previous results showing that in other teleosts, such as rainbow trout^20^, common carp^15^, hybrid striped bass^16^, sea bream^17^, and grass carp^18^, TGF-*β1* is expressed in all detected tissues. This suggests that the TGF-*β1* molecule plays multiple roles in teleost physiological activities, which has been proven in mammals. However, the *cTGF-β1* molecule showed the highest expression levels in the thymus, spleen and head kidney. Similar results were also observed in the grass carp, in which the expression of TGF*-β1* was the highest in those three tissues^18^. Because the thymus, spleen and head kidney tissues have been shown to be the major lymphoid organs in fish^21^, these results suggested that the TGF-*β1* molecule may predominantly play an immunological role in teleost physiological activities.

The TGF-*β* family members are ubiquitous molecules with pleiotropic functions in a wide variety of cells^22^,^23^,^24^. Notably, TGF-*β* is a key regulator of host defence that performs an essential role in immune function modulation in both the innate and adaptive immune pathways^5^,^25^,^26^. TGF-*β1*, the most important isoform of TGF-*β*, has been widely studied in mammals over the past few decades. However, information regarding the functional role of TGF-*β1* in fish immunity has been limited. In the present study, the effect of TGF-*β1* on the mRNA expression of the pro-inflammatory cytokines TNF-*α* and IL-*1β* in the absence or presence of LPS was determined in culter PBLs. The results showed that the *cTGF-β1* significantly increased the mRNA levels of TNF-*α* and IL-*1β*, but significantly attenuated the LPS-enhanced mRNA levels of these two cytokines. This finding was similar to the results found in mammals, in which the cytokine TGF-*β1* performs a dual role in the inflammatory reaction^9^,^27^. Mammalian TGF-*β*, which has been shown to be one of the first agents to appear in the inflammatory response, acts as a pro-inflammatory cytokine to trigger monocyte recruitment and cytokine expression^1^,^5^. At the end of inflammation, TGF-*β* functions as a terminal signal to turn off the inflammatory reaction^28^. Actually, parallel results have also been observed in several fish species in recent years. Jang *et al.* reported that mammalian *rTGF-β* could enhance or suppress trout macrophage respiratory burst activity^29^ under different conditions. In goldfish, TGF-*β1* significantly blocks the TNF-*α*-induced activation of macrophages and induces the proliferation of the goldfish fibroblast cell line CCL71^3^. In grass carp, TGF-*β1* down-regulates the LPS/PHA-stimulated proliferation of PBLs, which is in contrast to the stimulatory effect of TGF-*β1* alone in the same type of cells^30^. In red seabream, recombinant TGF-*β1* could induce HKL and PBL migration in a dose-dependent manner, but suppressed LPS-activated HKL and PBL migration^31^. These findings suggest that the teleost TGF-*β1* molecules may be employed in an immunoregulatory mechanism involved in the inflammation response that is analogous to the same system in mammals.

Regrettably, these previously mentioned studies that were conducted in fish species mostly focused on the interpretation of the TGF-*β1* immunoregulatory mechanism at the cellular level *in vitro*. Few works have been concerned with the immunological characteristics of this cytokine in organic tissues *in vivo*. The organ-based immune system is extremely sophisticated, and the constituent components of this system are ubiquitous and systemically acted upon during a single immune event. Aiming to assess the immune role of teleost TGF-*β1 in vivo*, the expression of this cytokine in culter thymus and spleen tissues after induction with poly I:C was examined. In the previous *in vitro* studies, teleost cells such as orange spotted grouper HKLs^32^ and teleost epithelial cells^33^ were also induced with poly I:C and showed obvious up-regulation in TGF-*β1* expression. Remarkably, despite the significant increase in *cTGF-β1* expression observed in the thymus and spleen, the time to peak TGF-*β1* expression was noticeably different in different teleost cell lines and organ tissues; TGF-*β1* expression peaked significantly earlier in the cell lines than in the organ tissues (hour 4 after stimulation in orange spotted grouper head kidney lymphocytes and hour 6 in teleost epithelial cells *vs* hour 72 after stimulation in culter thymus and spleen tissues), suggesting that the *cTGF-β1* molecule may synergistically act with other immunologic factors after the poly I:C induction *in vivo*. Overall, these results suggest that TGF-*β1* plays an immunoregulatory role in culter and that the teleost TGF-*β1* may be involved in a more complicated signalling pathway in response to exogenous infection *in vivo* compared to *in vitro*.

Interestingly, the *cTGF-β1* molecule in the thymus appeared to be more sensitive to poly I:C than in the spleen. The thymus is considered to be the central lymphoid organ^34^ and is primarily responsible for T cell development in teleosts^35^. As reported, the thymus controls the differentiation of the head kidney and spleen during the histogenesis of the lymphoid organs^25^,^36^. In rainbow trout, developing B cells mature in the head kidney and then migrate to sites of activation, which are either the spleen or the posterior kidney^37^. Accordingly, we supposed that the culter TGF-*β1* originated from the lymphocytes of the thymus and then migrated to the secondary immune organs, such as the head kidney and spleen.

Taken together, the results presented in this study may provide valuable information regarding the potential functional mechanism of TGF-*β1* in teleosts.

## Methods

### Animal uses

Healthy *C. alburnus* specimens were obtained from the Chidonghu fishery, Hubei Province, China and were maintained at the Aquatic Facility of the Fishery college, Huazhong Agricultural University. All animal use and studies were approved by the Institutional Animal Care and Use Committee of Hubei province, Wuhan, P. R. China. All procedures were carried out in accordance with the approved guidelines.

### Sample collection and nucleic acid extraction

All of the fish were approximately one year old and ranged from 100–150 g. The fish were kept at 25 °C in a flowthrough water system for two weeks before treatment. During the experiment, the fish were fed with commercial feed.

The heart, intestine, eye, head kidney, brain, spleen, muscle, liver, thymus, ovary and gill were removed from 8 freshly killed culter fish using a lethal dose of MS-222 at 200 mg/L and frozen immediately in liquid nitrogen and stored at −80 °C until RNA extraction.

The total RNA was extracted from the frozen tissues using TRIzol reagent (Life Technologies, USA) following the manufacturer’s instructions in combination with DNase I treatment. The RNA quality was assessed by running the samples on 1% agarose gels and visualizing by staining with ethidium bromide. The RNA concentration was measured using a NanoDrop 2000 spectrophotometer (NanoDrop Technologies, Wilmington, DE, USA) with an absorption at 260 nm.

### Amplification of c*TGF-β1* cDNA

Two pairs of degenerate primers (T1 and T2, Table 1) for amplification of the *cTGF-β1* cDNA partial sequence were designed at the conserved regions of teleost TGF-β*1* cDNA using the Primer Premier 5.0 software (Premier Biosoft International, USA). Reverse transcription polymerase chain reaction (RT-PCR) was performed using the total RNA from *C. alburnus* spleen as the template.

The first-strand cDNAs were synthesised using the RevertAid^TM^ First Strand cDNA Synthesis Kit (Fermentas, Canada) and oligo(dT)_18_ primers. PCR was carried out with 2 μl synthesized cDNA in a 50-μl reaction volume containing 10 pmol of each specific primer and 2 units of Taq DNA polymerase (Takara). The PCRs were all performed on a Veriti thermocycler (Life Technologies, USA). The PCR conditions were as follows: an initial denaturation of 94 °C for 5 min; 35 cycles of denaturation at 94 °C for 30 s, annealing for 30 s, and extension at 72 °C for 60 s; and a final extension at 72 °C for 7 min. The PCR products were detected on a 1.5% agarose gel and were then subcloned into pMD18-T vectors (Takara). The confirmed recombinant plasmid DNA was used as the template for automated sequencing using an ABI 3730 automated DNA sequencer. The nucleotide sequences of the sense strands of the plasmid inserts of three independent clones were determined and were then assembled using the CAP3 online software (http://pbil.univ-lyon1.fr/cap3.php).

To obtain the 5′ and 3′ ends of *cTGF-β1* cDNA, a number of transcript-specific sense or antisense primers (5′RACE and 3′RACE, Table 1) were designed from the partial cDNA sequence. The 5′- and 3′-rapid amplifications of the cDNA ends (5′- and 3′-RACE) were performed using the RACE cDNA Amplification Kit (Life Technologies, USA) with splenic total RNA as the template according to the manufacturer’s protocol. The nested PCR products were subcloned and sequenced as described above. Following the generation of the 5′- and 3′-RACE fragments, the core fragments were assembled to obtain the *cTGF-β1* cDNA full length.

### Sequence analysis and phylogenetic relationships

The nucleotide and amino acid sequences were identified using the BLAST program (GenBank, NCBI). Conserved TGF-*β1* motifs were identified using the analytical tools provided by the ExPASy Molecular Biology Server (http://www.expasy.org). Multiple alignments were performed using ClustalX 1.83^38^. Phylogenetic relationships were investigated using the Neighbour-Joining (NJ) method in the Mega 5.0 program (Molecular Evolutionary Genetic Analysis)^39^. A bootstrap analysis was conducted using 10,000 replicates.

### Construction of the *pET32a- cTGF-β1* expression vector

Culter TGF-*β1* constructs were designed for expression using the pET32a prokaryotic expression system. The culter TGF-*β1* transcript encoding the mature peptide portion of the predicted amino acid sequence was amplified by PCR using the primers T-y (Table 1). PCR was conducted on a Veriti thermocycler with Taq DNA polymerase (Takara). The amplification procedure consisted of 35 cycles of 94 °C for 30 s, 58 °C for 30 s, and 72 °C for 60 s, followed by a final extension at 72 °C for 7 min. The PCR product was digested with BamHI and XhoI and then cloned into the pET-32a vector to express a fusion protein of *cTGF-β1* with an N-terminal 6 × His tag. This recombinant plasmid was sequenced and named *pET32a-cTGF-β1*.

### Production, purification and analysis of recombinant culter *TGF-β1*

The *pET32a-cTGF-β1* vector was transformed into BL21 competent cells (Life Technologies) to express the fusion protein. The bacteria were inoculated in LB media with vigorous shaking at 37 °C. The bacterial optical density was measured using a NanoDrop 2000 spectrophotometer with absorption at 600 nm every two hours. When the OD600 reached approximately 0.6, the protein expression was induced by isopropyl-beta-D-thiogalactopyranoside to a final concentration of 1.0 mM. Eight hours after induction under the previously described conditions, the bacteria were harvested by centrifugation at 5000 × *g* for 15 min and resuspended with binding buffer. Then, the soluble protein was purified using the 6 × His-Tagged Protein Purification Kit (Cwbio, China) according to the manufacturer’s specifications. The removal and monitoring of endotoxin residues were achieved using the ToxinEraser^TM^ Endotoxin Removal Kit and the ToxinSensor^TM^ Chromogenic LAL Endotoxin Assay Kit (GenScript), respectively, following the user manuals. The expression of the 6 × His-Tagged *cTGF-β1* was confirmed on a 12% sodium dodecyl sulfate polyacrylamide gel electrophoresis (SDS-PAGE) and was visualized by staining with Coomassie brilliant blue. The protein concentration was measured using a NanoDrop 2000 spectrophotometer with an absorption at 280 nm.

A western blot assay was conducted to identify the fusion protein. Briefly, the purified sample was separated by SDS-PAGE using a 12.5% polyacrylamide gel and transferred to a nitrocellulose membrane (Life Technologies, USA) at 150 mA for 1.5 h in transfer buffer. The membrane was incubated overnight at 4 °C in the presence of anti-6 × His mouse antibody (Life Technologies, USA) after blocking with 5% (w/v) nonfat dry milk. The membrane was subsequently washed with TBST buffer, incubated with AP-goat anti-Mouse IgG and developed using NBT-BCIP.

### Isolation of culter peripheral blood leukocytes (PBLs)

Blood samples were obtained from the caudal vein of culter using a heparinized syringe. The blood samples were then diluted with an equal volume Hanks’ balanced salt solution and layered onto a Percoll (Sigma) discontinuous density gradient of 60%/67%. The samples were then centrifuged at 400 × *g* for 30 min at 4 °C. The cell layers at the interphase were transferred to another tube, an equal volume of Hanks’ solution was added, and the samples were centrifuged at 200 × *g* for 10 min at 4 °C. The cells were collected, counted using a haemocytometer under a light microscope, and seeded in a 24-well plate at 5 × 10^6^ cells/well.

### Drug treatment assay

Drug treatments were performed using different test substances, including lipopolysaccharide (LPS) (10 μg/ml) (Sigma-Aldrich) and recombinant culter TGF-*β1* (100 ng/ml). After treatment with the drug for 6 hours, the cells were collected from the culture plates, centrifuged (10,000 × *g* for 1 min) and immediately frozen until further use. In the control, no inducer was added.

To assess the role of the TGF-*β1* molecule in the culter immunity *in vivo*, an intraperitoneal injection experiment was conducted, as previously described^40^. Briefly, eighty fish were equally divided into two groups: in one group, each fish was induced with approximately 2.5 mg/kg fish poly I:C; in the other group, each fish was injected with the same volume of 0.65% physiological saline as the control. Four fish from each group were euthanized at 0 h, 4 h, 12 h, 24 h, 48 h, 72 h, 96 h and 120 h after injection. At each time point, the spleen and thymus samples were dissected and frozen.

### qPCR and expression analysis

The specific primers for qPCR of TGF-*β1*, TNF-*α* and IL-*1β* in the topmouth culter were designed using Primer Premier 5.0. To verify the specificity, the primers were first tested in a normal PCR amplification. The PCR products were visualized on a 2% agarose gel. The qPCR were carried out in a final volume of 10 μl consisting of 1 × Platinum^®^ SYBR^®^ Green qPCR SuperMix-UDG (Life Technologies), 0.2 μM of each primer, and 10 ng of cDNA template. The reactions were run in triplicate. The ABI PRISM 7500 HT platform (Life Technologies, USA) was used for all real-time assays. The reactions were initially denatured at 95 °C for 10 sec, followed by 40 cycles of 95 °C for 10 sec and annealing (60 °C for TGF-*β1* and IL-*1β*, 62 °C for TNF-*α*) 30 sec. A melting curve analysis was performed at the end of assay to assess the amplification specificity. The relative expression levels were measured using the 2^−ΔΔ*Ct*^ method^41^ with *β*-action as an internal reference. Statistical significance of the data was analysed using a one-way ANOVA, and the results were deemed to be significant at P ＜ 0.05.

## Additional Information

**How to cite this article**: Qi, P. *et al.* Dissecting the role of transforming growth factor-β1 in topmouth culter immunobiological activity: a fundamental functional analysis. *Sci. Rep.* **6**, 27179; doi: 10.1038/srep27179 (2016).

## Supplementary Material

Supplementary Information

## Figures and Tables

**Table 1:** Sequence of primers used for amplification of partial cDNA, 3′ and 5′ RACE of topmouth culter TGF-*β1* and qPCR analysis for cTGF*-β1*,TNF*-α* and IL*-1β*.

Primer	Sequence (5′–3′)	Usage
T-1	GAYCTGGRNTGGAARTGGAT	TGF*-β1* partial
	GARGCBATTMGRRGHCAGAT	
T-2	GCMGAKGCWCCDGGRTTGTG	TGF*-β1* partial
	GTTTTCAGCATTCCAGATGTAGGTG	
5′RACE	CCTTGTGTTGTCTTCC	TGF*-β1* 5′RACE
	GACTGCAGGTACGCAACATGG	
	GAGTATTTGTTCTCAGCATTCC	
3′RACE	GTGCATCTGCTCAGCCATGTTGCGTACCT	TGF*-β1* 3′RACE
	CCTGCAGTCCTCGACCCTCTACCAATTCT	
T-y	CAGGATCCGATGTTGGCACTGATGTAACTTG	TGF*-β1* expression
	GACCTCGAGACTGCACTTGCAGCTCCTCAC	
T-q	GGAGGTGCACAAGTTCATTAT	TGF*-β1* qPCR
	GGCAGATATGATGGTGAAGCC	
TNF-q	ACTGTCTCCTTCACGCTCCA	TNF-*α* qPCR
	CTGTGAGGTGCCATTCGCTT	
IL-q	ACGCAGGCCGGAGCCTCTGT	IL-*1β* qPCR
	CCTGTAGATGAGGCGGCTGTC	
*β*-actin	TGCGTGACATCAAGGAGAAG	internal control
	GCTGGAAGGTGGACAGAGAG	
