# Supplementary material for: Dissecting the role of transforming growth factor-β1 in topmouth culter immunobiological activity: a fundamental functional analysis
Source: Sci Rep. 2016 Jun 2;6:27179. doi: 10.1038/srep27179 (PMC4890032; doi:10.1038/srep27179)
Supplement: Supplementary Information [file srep27179-s1.pdf]

Dissecting the role of transforming growth factor-beta1 in topmouth culter immunobiological activity: a fundamental and functional analysis

Pengzhi Qi[a, *], Congxin Xie[b], Baoying Guo[a], Changwen Wu[a]

[a] *National Engineering Research Center of Marine Facilities Aquaculture, Marine Science and Technology College, Zhejiang Ocean University, Zhoushan 316004, China*

[b] *College of Fisheries, Huazhong Agricultural University, Wuhan 430070, China*

[*] Corresponding author. Tel.: +86 580 2554818 Fax: +86 580 2554818

E-mail address: qpz2004@sina.com

Fig S1. Nucleotide and deduced amino acid sequence of *cTGF-β1*. The GenBank accession number is KJ704192.

Fig S2. The SDS-PAGE analysis for the expression *in vitro of* the *cTGF-β1* mature peptide portion. M, protein molecular weight marker; lane 1, 4-6, lysates of transformed bacteria after induction with IPTG; lane 2, 3 and 7, lysates of transformed bacteria of non-inserted control.

Fig S3. Western blot analysis of recombinant *cTGF-β1* using anti-6×His tag mouse antibody. M, protein molecular weight marker; lane 1-6, the expression of recombinant *cTGF-β1* mature peptide portion.

```
   1 acggaggccatgcgcgtgcctgggaaggtgtgtggtccgggggcc
  46 gcgtcagattctttctttccctggcgcgggtgaggaaccgcgccc
  91 gaggcagtcgggggtacgtggcgttaaacgcgacagggatctagt
 136 ttccgacagacatgtaatgcttacatatatctatgttcaagtcag
 181 cactaggccgtgttcaggtatacactagtaattgcatggtcttca
 226 ctaagtttgtttatatgggacaggggttgctccatttggccaaac
 271 tgcactgcactgtctggatggaactctgaaacagatgatgtgtca
 316 ggaatttcccatattagaaagacatcagctgttctgttggctacg
 361 tattcaaggtgctttccagaatcatcatcaggtagcatcacctc
 406 cataaccagcaacactactgtgtttgtttgatccagacgctctac
 451 gtcaattacagactctttctgaacctctctccttacttaaatctg
 496 ttctaaaagacagtttgggctcagatttggcacc
 530 atgaggtttgtttgcttagtactgacggccctgtgcatggtggct
     M  R  F  V  C  L  V  L  T  A  L  C  M  V  A
 575 ggaacgggcagtatgtccacctgcaagactctggacctggaggtg
     G  T  G  S  M  S  T  C  K  T  L  D  L  E  V
 620 gtgaagaagaaacggatagaggctatccgcggtcagatcctcagc
     V  K  K  K  R  I  E  A  I  R  G  Q  I  L  S
 665 aaattgcgcatggtgaaagagcctgaatcggagatagacgatgtt
     K  L  R  M  V  K  E  P  E  S  E  I  D  D  V
 710 ggacaaaagatcccagattccttgctttctttgtacaacagcact
     G  Q  K  I  P  D  S  L  L  S  L  Y  N  S  T
 755 gttgaactgagcgaagaaatgaagctgaagactgtccctctgcag
     V  E  L  S  E  E  M  K  L  K  T  V  P  L  Q
 800 gctgaagatgaggactactttggcaaggaggtgcacaagttcatt
     A  E  D  E  D  Y  F  G  K  E  V  H  K  F  I
 845 atccggcaagctcagaatggcacaaagcatcagatgtttttaat
     I  R  Q  A  Q  N  G  T  K  H  Q  M  F  F  N
 890 gtatcagagatgagacagagtattccagactaccggctgctgtct
     V  S  E  M  R  Q  S  I  P  D  Y  R  L  L  S
 935 caagcagagttacggcttcggattaagaatcccaccatggaccaa
     Q  A  E  L  R  L  R  I  K  N  P  T  M  D  Q
 980 gagcagaggctggaactgtatcgtggagtaggtgatcaggctcga
     E  Q  R  L  E  L  Y  R  G  V  G  D  Q  A  R
1025 tatctgggcacccgctttgtctccaaggattggtccaatcgctgg
     Y  L  G  T  R  F  V  S  K  D  W  S  N  R  W
1070 ctctcgtttgatgtgaaacagacgatgacagaatggctgcagagt
     L  S  F  D  V  K  Q  T  M  T  E  W  L  Q  S
1115 tcagaagatgaagaaagtttagaactgaggttgtactgtggctgt
     S  E  D  E  E  S  L  E  L  R  L  Y  C  G  C
1160 aagacagaccagcagagtacagataaattcctctttactatatca
     K  T  D  Q  Q  S  T  D  K  F  L  F  T  I  S
1205 ggactggacaaacagagaggcgacactgccagtctggcagatatg
     G  L  D  K  Q  R  G  D  T  A  S  L  A  D  M
1250 atggtgaagccttacattttggctttatctttgccctctaacggc
     M  V  K  P  Y  I  L  A  L  S  L  P  S  N  G
1295 aactcccttgcatccggtcgcagtaaacgagatgttggcactgat
     N  S  L  A  S  G  R  S  K  R  D  V  G  T  D
1340 gtaacttgtgatgaaaaaactgagacctgttgtatgcgtaagctt
     V  T  C  D  E  K  T  E  T  C  C  M  R  K  L
1385 tatattgactttcggaaagatctgggctggaagtggatccacaag
     Y  I  D  F  R  K  D  L  G  W  K  W  I  H  K
1430 cccaaaggatactatgctaactactgcatgggctcctgcacctac
     P  K  G  Y  Y  A  N  Y  C  M  G  S  C  T  Y
1475 atctggaatgctgagaacaaatactctcagatattagccttgtac
     I  W  N  A  E  N  K  Y  S  Q  I  L  A  L  Y
1520 aaacaccacaaccctggtgcatctgctcagccatgttgcgtacct
     K  H  H  N  P  G  A  S  A  Q  P  C  C  V  P
1565 gcagtcctcgaccctctaccaattctttactatgtgggaagacaa
     A  V  L  D  P  L  P  I  L  Y  Y  V  G  R  Q
1610 aacaaggtggagcagttgtccaacatggtggtgaggagctgcaag
     N  K  V  E  Q  L  S  N  M  V  V  R  S  C  K
1655 tgcagttaa 1663
     C  S  *
1664 agtatgactatataacattattttatttcagaaagtgctccatgg
1709 cactatctctctctggacgattagtaagcaaacacacagatagat
1754 gaacatataaagtatcactgggaaggagaatcaggaactgagaca
1799 actgacaaggatgggaaaaggagaaaggagagtcttgtaaaaaaa
1844 ataggagggaaaattaaacaggggaataaaaaatatctttgaaaa
1889 tgagactttctcagccatgcccctctacagtggcacaagtgcaca
1934 caaaggcattccgttaaagcacttcacaaaacaagctcaatatca
1979 aaccaaatatatgctttgttatctttttatgtttatttacatta
2024 ctcattcccattaatttattgtgcttgttaagttcataatttgat
2069 tataccatgagggtagtcgtgtagcattgcatgccaaatgtacaa
2114 tgaagatttctaatggtgctgtactgataaaggaaatgatgtcaa
2159 aaaaaaaaaaaaaaaaa
```

Fig S1. Nucleotide and deduced amino acid sequence of *cTGF-β1*. The GenBank accession number is KJ704192.

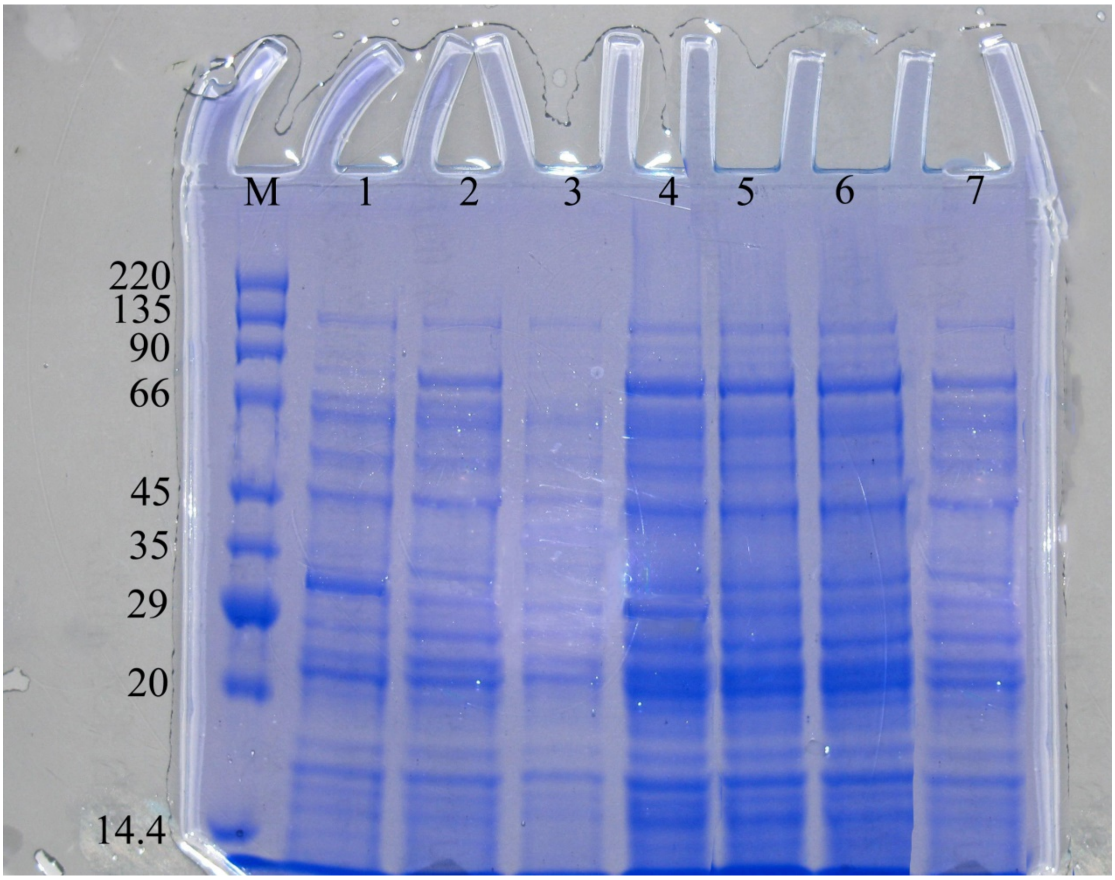

Fig S2. The SDS-PAGE analysis for the expression *in vitro of* the *cTGF-β1* mature peptide portion. M, protein molecular weight marker; lane 1, 4-6, lysates of transformed bacteria after induction with IPTG; lane 2, 3 and 7, lysates of transformed bacteria of non-inserted control.

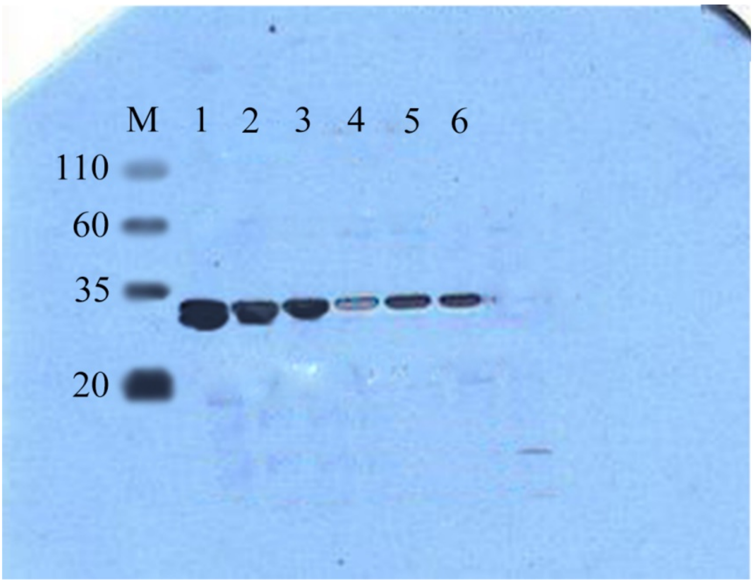

Fig S3. Western blot analysis of recombinant *cTGF-β1* using anti-6×His tag mouse antibody. M, protein molecular weight marker; lane 1-6, the expression of recombinant *cTGF-β1* mature peptide portion.
